# On the development of survey methods for novel mean imputation and its application to abalone data

**DOI:** 10.1016/j.heliyon.2024.e31423

**Published:** 2024-05-20

**Authors:** Syed Abdul Rehman, Javid Shabbir, Laila A. Al-essa

**Affiliations:** a Department of Statistics, Quaid-i-Azam University, Islamabad, 45320, Pakistan; b Department of Statistics, University of Wah, Wah Cantt, 47040, Pakistan; c Department of Mathematical Sciences, College of Science, Princess Nourah bint Abdulrahman University, P.O. Box 84428, Riyadh, 11671, Saudi Arabia

**Keywords:** Order statistics, Missing data, Mean imputation, Imperfect ranking, Relative efficiency

## Abstract

Non-response in surveys is a common problem faced by surveyors, this results in missing data. Missing values are often omitted when doing any statistical analysis, but this reduces the sample size and consequently decreases the precision of estimates. In such situations, imputation is a commonly used method to deal with missing data, this involves estimating the missing values based on the observed data. In this paper, we propose two new estimators for the finite population mean, formulated using two suggested sampling methods and their associated imputation strategies. We derive the variance of the proposed estimators and obtain conditions under which these estimators are more efficient than existing estimators. We conduct a simulation study to assess the relative efficiency (RE) of the proposed estimators for varying sample sizes, response rates, and ranking criteria. For real-world application, we consider data on measuring the characteristics of abalone. The simulation results demonstrate that the proposed mean estimators based on the suggested imputation methods are more efficient than the existing methods in estimating the mean of the finite population.

## 1 Introduction

Estimation of population parameters using information obtained in a sample is an important statistical technique. In the literature, several methods of sampling are available for the collection of a sample from an underlying population. Simple random sampling (SRS) is an easy and technically straightforward sampling method, but the estimates based on it are not very precise. To estimate population parameters more precisely, the literature can provide other sampling methods, including ranked set sampling (RSS). This sampling method is useful in situations where collecting information about the study variable is expensive and time-consuming; however, the study variable can easily be ordered based on the characteristics of a closely related auxiliary variable. This method involves examining a larger number of sampling units based on their relative size and subsequently selecting a small subset from this pool. This way, RSS allows for increased precision in estimates by decreasing the sampling error. An advantage of RSS is that, if non-response occurs on the study variable, it can still be ordered based on the ranks of a closely related auxiliary variable that does face non-response. The RSS technique was initially introduced by [12], where units of the study variable were ranked using the surveyor's judgments simply. [25] suggested an unbiased estimator of the population under RSS. The ranks of the auxiliary variable were used to order the units of the study variable by [10] and [23]. Several authors claim that the use of auxiliary variables for ranking can produce more efficient results since it decreases the sampling variation. This claim is supported by the work of [7], [11], [24], [1], and [13].

On the other hand, non-response is a critical problem faced by surveyors, which refers to the inability to obtain responses from one or more units of the population. Non-response is very common in primary data collection in many subject areas, especially in surveys related to farmers and agriculture. Non-response in surveys may arise from the respondent's privacy concerns or time limitations faced by the surveyors. Other causes of non-response include inadequately designed surveys, technological obstacles, demographic factors, and the sensitivity of the questions being asked. Non-response causes missing data, resulting in gathering a sample that does not represent the population sufficiently, potentially causing bias in estimates. [19] classified missing values into three types, i.e., Missing Completely at Random (MCAR), Missing at Random (MAR), and Not Missing at Random (NMAR). MCAR assumes that missing data occurs randomly without any systematic pattern, whereas MAR considers that missing data occurs randomly, but the probability of missingness may depend on observed data. NMAR assumes that missing data occurs in a non-random pattern, depending on both observed and unobserved data. A comparison of MCAR and MAR in missing data can be seen in [9] and [5]. [20] and [22] highlighted that failure to address missing data can lead to imprecise conclusions, especially when there are more than 5% missing values in the data. [26] examined the issue of missing data and compared the estimation results. A variety of approaches are employed to address missing data. List-wise and pair-wise deletion are conventional methods; however, they reduce the sample size. Other approaches are sub-sampling and the imputation of the sample mean or median. Mean imputation is a fundamental and widely used technique to handle non-response, it ensures the usability of a data set for analysis by maintaining sample size and retaining the statistical power of the analysis. Various approaches on imputation are available in the literature. Numerous authors have suggested different techniques for mean imputation under SRS for the estimation of population parameters. The work of [2] and [8] can be seen in this regard. While mean imputation is a fundamental imputation technique, it serves as the foundation for more advanced techniques like ratios, regression, and multiple imputation. [16], [17] and [21] have proposed robust regression methods for mean imputation, while mean imputation through quantile regression is proposed by [6] and [18] respectively. A comparison of mean imputation, hot deck imputation, k-nearest neighbors imputation (KNN), and stochastic regression imputation presented by [3]. The issue of missing values under the RSS technique was studied by [4], who proposed several efficient methods of mean imputation.

Modern data often have complex structures, like hierarchical or longitudinal. Traditional imputation methods may not adequately handle these complexities, so newly designed imputation techniques are required to address the evolving challenges presented by modern datasets. Inspired by [4], we apply the theory of RSS to present advances in the mean imputation and propose two novel methods of sampling when the undergoing survey anticipates non-response. Based on these methods, we then propose two efficient estimators of the finite population mean. In our study, we assume that the missing data occurs completely at random. Our objective is to provide advanced and efficient methods of mean imputation that can yield more precise estimates of the finite population mean. This study begins by introducing the theory and mathematics of RSS in Section 2, followed by Section 3, where the existing methods of imputation are discussed. Section 4 presents the suggested imputation methods, presenting both the algorithm and the sampling scheme. An efficiency comparison between the proposed estimators and existing ones is also provided at the end of Section 4. The simulation procedure and its findings are discussed in Section 5, while Section 6 provides an application of the proposed estimators to real-life data. In Section 7, this study is summarized with concluding remarks and recommendations.

## 2 RSS procedures

In this section, we describe the procedures for selecting a sample using the RSS and MRSS methods. Estimators of the population mean and their corresponding variance expressions are provided for the two RSS methods.

### 2.1 The classical RSS (RSS)

The procedure for selecting a sample using RSS is as follows.

Initially, we identify $\nu^{2}$ units of the population and assign them to *ν* independent sets, each of size *ν*. In each set, rank the units using some visual judgments. Choose the first order statistic from the first set, while the second order statistic is selected from the second set. Keep choosing units in this manner until the $\nu^{th}$ order statistic is chosen from the $\nu^{th}$ set. The selected sample can be represented as ${\left(Y_{i\left(i\right)j}\right)}_{i=1}^{\nu }$, where j=1,2,...r represents the number of times an RSS procedure is repeated to obtain a final sample of size n=rν.

The mean estimator under this method was developed by [25], given by

(1) $${\overset{¯}{y}}_{rss}=\frac{1}{r\nu }\sum_{j=1}^{r}\sum_{i=1}^{\nu }Y_{i(i)j}.$$

The estimator ${\overset{¯}{y}}_{rss}$ is unbiased, whereas its variance is given by

(2) $$V\left({\overset{¯}{y}}_{rss}\right)=\frac{\sigma^{2}}{r\nu }-\frac{1}{r\nu^{2}}\sum_{i=1}^{\nu }\Delta_{{}_{\left(i\right)}}^{2},$$

where $\Delta_{\left(i\right)}=\mu_{\left(i\right)}-\mu$ shows the deviation of $i^{th}$ order statistics mean $\mu_{\left(i\right)}$ from the mean of the total population *μ*, and $\frac{\sigma^{2}}{r\nu }$ is the variance of the mean estimator under the SRS method. The Equation (2) shows that RSS is at least as efficient as SRS.

### 2.2 Median RSS (MRSS)

This approach of RSS was proposed by [14]. It is more suitable when the population being studied is not symmetrical. The procedure for selecting a sample through MRSS is as follows:

Initially, examine $\nu^{2}$ units from population, distribute them into *ν* independent sets each of size *ν* and rank the units in each set.

• If *ν* is odd, then select the median i.e., ${\left(\frac{\nu +1}{2}\right)}^{th}$ unit from all sets. The selected sample can be represented as $Y_{i\left(\frac{\nu +1}{2}\right)j}$; j=1,...,r and i=1,...,ν.
• If *ν* is even, then select ${\left(\frac{\nu }{2}\right)}^{th}$ unit from first $\left(\frac{\nu }{2}\right)$ sets, whereas ${\left(\frac{\nu +2}{2}\right)}^{th}$ unit is selected from the remaining sets. The selected sample can be expressed as $Y_{1\left(\frac{\nu }{2}\right)j}$, $Y_{2\left(\frac{\nu }{2}\right)j}$,..., $Y_{\frac{\nu }{2}\left(\frac{\nu }{2}\right)j}$, $Y_{\frac{v+2}{2}\left(\frac{\nu +2}{2}\right)j}$,..., $Y_{\nu \left(\frac{\nu +2}{2}\right)j}$.

The mean estimator based on MRSS for odd and even sample size is given by

$${\overset{¯}{y}}_{mrss}=\left\{ \begin{array}{cc} \frac{1}{r\nu }\sum_{j=1}^{r}\sum_{i=1}^{\nu }Y_{i\left(\frac{\nu +1}{2}\right)j}, & \;\text{if}\;\text{ν}\;\text{is odd}\\ \frac{1}{r\nu }\sum_{j=1}^{r}\left(\sum_{i=1}^{\frac{\nu }{2}}Y_{i\left(\frac{\nu }{2}\right)j}+\sum_{i=\frac{\nu +2}{2}}^{\nu }Y_{i\left(\frac{\nu +2}{2}\right)j}\right), & \;\text{if}\;\text{ν}\;\text{is even} \end{array}\right.$$

Mean estimator under MRSS is unbiased in both cases whereas its variance is given by

(3) $$V\left({\overset{¯}{y}}_{mrss}\right)=\left\{ \begin{array}{cc} \frac{\sigma^{2}}{r\nu }-\frac{1}{r\nu }\sum_{i=1}^{\nu }{\left(\mu_{\left(\frac{\nu +1}{2}\right)}-\mu_{y}\right)}^{2}, & \;\text{if}\;\text{ν}\;\text{is odd}\\ \frac{\sigma^{2}}{r\nu }-\frac{1}{r\nu^{2}}\left(\sum_{i=1}^{\frac{\nu }{2}}\Delta_{\left(\frac{\nu }{2}\right)}^{2}+\sum_{i=\frac{\nu +2}{2}}^{\nu }\Delta_{\left(\frac{\nu +2}{2}\right)}^{2}\right), & \;\text{if}\;\text{ν}\;\text{is even} \end{array}\right.$$

Equation 3 shows that the MRSS is more efficient than SRS.

### 2.3 RSS with imperfect ranking

In practice, the visual judgment of the study variable is not always accessible. This problem was solved by [10] who introduced the use of a closely related auxiliary variable *X* to order the values of the study variable *Y*. This method was termed imperfect ranking, and it might involve errors in ranking, especially when the correlation coefficient between the two variables is not sufficiently high. In this case, the study variable is denoted by $Y_{\left[i\right]}$. Following [10], it is assumed that (Y,X) follows a bivariate normal distribution and that the regression of *Y* in *X* is linear, that is,

(4) $$Y_{i[i]j}=\mu_{y}+\rho_{yx}\frac{\sigma_{y}}{\sigma_{x}}\left(X_{i(i)j}-\mu_{x}\right)+e_{ij},$$

where $\rho_{yx}$ is the coefficient of correlation between *Y* and *X*, and $e_{ij}$ is the error term with zero expected value and unit variance, i.e., $V(e_{ij})=\sigma^{2}\left(1-\rho_{yx}^{2}\right)$. The term $X_{i(i)j}$ denotes the $i^{th}$ order statistics in the $i^{th}$ set of the $j^{th}$ cycle. The corresponding judgment order statistics of the study variable to $X_{i(i)j}$ are denoted by $Y_{i[i]j}$. Expressions for the mean estimator and its variance are given by

(5) $${\overset{¯}{y}}_{rss}=\frac{1}{r\nu }\sum_{j=1}^{r}\sum_{i=1}^{\nu }Y_{i[i]j}$$

and

(6) $$V\left({\overset{¯}{y}}_{rss}\right)=\frac{\sigma^{2}}{r\nu }-\frac{1}{r\nu }\sum_{i=1}^{\nu }\Delta_{\left[i\right]}^{2}.$$

In the case of perfect ranking, it is possible that units of the study variable are visually ordered, but one or more units fail to respond among them. In the case of imperfect ranking, we assume that the study variable is ordered based on the ranks of the auxiliary variable, for which complete information is available from all respondents, however, non-response only occur on the study variable.

## 3 Existing imputation methods

This section reviews some existing methods of imputation that are currently employed for the imputation of missing values. The procedures for each method are explained, along with their respective mean estimators and variances.

### 3.1 The naive approach

Let the population Ψ of size *M* is under study and we draw a sample *s* of size *m* by the method of SRSWOR to estimate the population mean. Let $m_{1}$ units respond to the survey, while $m_{2}=m-m_{1}$ units fail to respond, these are classified as missing data. Let us denote the set of responding units by *R* and the set of missing units by $\overset{¯}{R}$. The values of $Y_{i}$ are observed for i∈R, while the missing values of $Y_{i}$ for $i\in \overset{¯}{R}$ are replaced by the mean of the responded units. This is called mean imputation, and the variable after imputation of missing values can be expressed as

(7) $$Y_{i}=\left\{ \begin{array}{cc} Y_{i} & \text{if }i\in R\\ {\overset{¯}{y}}_{m_{1}} & \text{if }i\in \overset{¯}{R} \end{array}\right.$$

where ${\overset{¯}{y}}_{m_{1}}={\left(m_{1}\right)}^{-1}\sum_{i=1}^{m_{1}}Y_{i}$ is mean of the responding units. Then estimator of the population mean under this method is

(8) $${\overset{¯}{y}}_{Isrs}=\left(\sum_{i\in R}Y_{i}+\sum_{i\in \overset{¯}{R}}{\overset{¯}{y}}_{m_{1}}\right)=\frac{1}{m_{1}}\left[m_{1}{\overset{¯}{y}}_{m_{1}}+\left(m-m_{1}\right){\overset{¯}{y}}_{m_{1}}\right].$$

The estimator ${\overset{¯}{y}}_{Isrs}$ is unbiased, whereas variance of ${\overset{¯}{y}}_{Isrs}$ is given by

(9) $$Var\left({\overset{¯}{y}}_{Isrs}\right)=\lambda_{m_{1}}\sigma^{2},$$

where

$\lambda_{m_{1}}={\left(Mm_{1}\right)}^{-1}\left(M-m_{1}\right)$.

### 3.2 Mean imputation under RSS

This method of mean imputation was proposed by [4] under the RSS method. This approach is slightly different from the naive method. This method can be described as:

Initially, draw a sample of size *ν* by the method of RSS as discussed in Section 2.1. Actual measurements are obtained from them and assume that only $\nu^{\prime }\left(<\nu \right)$ units of the study variable can provide a response, while the remaining $\left(\nu -\nu^{\prime }\right)$ units fail to respond. This procedure is repeated *r* times so that a final sample of size m=rν, among which the number of respondents is $m_{1}≅r\nu^{\prime }$ such that $m_{1}<m$. Let *P* show the probability that the $i^{th}$ unit in the RSS sample can provide the response, while (1−P) is the probability of failing to obtain a response in the sample *s*. The sample mean ${\overset{¯}{y}}_{B1}$ and its variance based on responded units are given by

(10) $${\overset{¯}{y}}_{B_{1}}=\frac{1}{r\nu^{\prime }}\sum_{j=1}^{r}\sum_{i=1}^{\nu^{\prime }}Y_{i\left(i\right)j}$$

and

(11) $$V\left({\overset{¯}{y}}_{B_{1}}\right)≅\frac{\sigma^{2}}{r\nu P}-\frac{1}{r{\left(\nu P\right)}^{2}}\sum_{i=1}^{\nu^{\prime }}\Delta_{\left(i\right)}^{2},$$

then the study variable after imputation of mean can be expressed as

(12) $$Y_{\cdot i}=\left\{ \begin{array}{cc} Y_{i(i)j} & \;\text{if }i\in R\text{ with probability }\text{P}\\ {\overset{¯}{y}}_{B_{1}} & \;\text{if }i\in \overset{¯}{R}\text{ with probability }(1-P) \end{array}\right.$$

Since *P* is probability of obtaining response, it follows that $E\left(r_{i}^{-k}\right)≅E{\left(r_{i}\right)}^{-k}$. This approach provides the unbiased estimator of the population mean and its variance as

(13) $${\overset{¯}{y}}_{Irss}=\frac{1}{m}\left(\sum_{j=1}^{r}\sum_{i=1}^{\nu^{\prime }}Y_{i\left(i\right)j}+\left(m-m_{1}\right){\overset{¯}{y}}_{B_{1}}\right)$$

and

(14) $$Var\left({\overset{¯}{y}}_{Irss}\right)≅\lambda_{m_{1}}\sigma^{2}-\frac{1}{r{\left(\nu P\right)}^{2}}\sum_{i=1}^{\nu^{\prime }}\Delta_{\left(i\right)}^{2}.$$

### 3.3 Mean imputation under MRSS

This method of imputation was suggested by [4] while keeping in view the efficiency of MRSS. This method is similar to the previous method, and its procedure is described as:

Initially, selected *ν* units from the population using the method described in Section 2.2. Let $\nu^{\prime }$ units respond to the survey, while $\left(\nu -\nu^{\prime }\right)$ units fail to respond. Repeat this process *r* times to obtain a final sample of size m=rν. The total number of responding units is $m_{1}=r\nu^{\prime }$, while the number of missing observations is $m-m_{1}$. The sample mean ${\overset{¯}{y}}_{B2}$ and its variance of responding units are given by

(15) $${\overset{¯}{y}}_{B_{2}}=\frac{1}{r\nu^{\prime }}\sum_{j=1}^{r}\sum_{i=1}^{\nu^{\prime }}Y_{i\left(med\right)j}$$

and

(16) $$V\left({\overset{¯}{y}}_{B_{2}}\right)≅\frac{\sigma^{2}}{r\nu P}-\frac{1}{r{\left(\nu P\right)}^{2}}\sum_{i=1}^{\nu^{\prime }}\Delta_{\left(med\right)}^{2}.$$

The term $Y_{i\left(med\right)j}$ is a general notation of the selected units for even and odd set size through MRSS procedure. The study variable after imputation of missing values can be expressed as

(17) $$Y_{\cdot i}=\left\{ \begin{array}{cc} Y_{i(med)j} & \;\text{if }i\in R\text{ with probability }\text{P}\\ {\overset{¯}{y}}_{B_{2}} & \;\text{if }i\in \overset{¯}{R}\text{ with probability }(1-P) \end{array}\right.$$

Where *P* is probability of obtaining response in sample *s*. The corresponding estimator of population mean is given by

(18) $${\overset{¯}{y}}_{Mrss}=\frac{1}{r\nu }\left(\sum_{i\in R}Y_{i\left(med\right)j}+\left(m-m_{1}\right){\overset{¯}{y}}_{B_{2}}\right).$$

The estimator ${\overset{¯}{y}}_{Mrss}$ is unbiased, whereas its variance is given by

(19) $$V\left({\overset{¯}{y}}_{Mrss}\right)=\left\{ \begin{array}{cc} \frac{1}{r\nu^{2}}\left(nP+\frac{\left(1-P\right)}{P}\right)\sigma_{\left(\frac{\nu +1}{2}\right)}^{2} & \;\text{if}\;\text{n}\;\text{is odd}\\ \frac{1}{4r\nu^{2}}\left\{\left(nP_{1}+\frac{1-P_{1}}{P_{1}}\right)\sigma_{\left(\frac{\nu }{2}\right)}^{2}+\left(nP_{2}+\frac{1-P_{2}}{P_{2}}\right)\sigma_{\left(\frac{\nu +2}{2}\right)}^{2}\right\} & \;\text{if}\;\text{n}\;\text{is even}, \end{array}\right.$$

where $P_{1}$ and $P_{2}$ shows the probability of obtaining response for observation $Y_{\left(\frac{\nu }{2}\right)}$ and $Y_{\left(\frac{\nu +2}{2}\right)}$, respectively, in case of even sample size. For more detail, see [4].

## 4 Proposed methods

The existing methods of mean imputation often use one value to impute all missing entries, which can reduce the variability in the datasets, potentially leading to an underestimate of the standard errors and confidence intervals. In contrast, we suggested mean imputation approach that utilize different values to impute missing observations based on their order statistics, ensuring to attain the actual variability in the data. This method is more like weighted imputation, where the imputation of a value depends on the order of the missing observation.

This section introduces two new methods for mean imputation, explaining the procedure of the proposed methods, presenting an algorithm for their implementation, and developing mean estimators based on these methods. Expressions for the variance of proposed estimators are derived and compared with existing estimators. Conditions under which the proposed mean estimator performs better are also given.

### 4.1 Proposed method I

This method employs the SRS technique to collect an initial sample of size n=rν, and then sorting sample units into *r* sets each of size *ν*. The procedure for selecting a sample and imputing the mean for missing values is described as follows:

Initially, examine a sample of m=r×ν units from the population and assign them to *r* independent sets, each of size *ν* units. Rank the units within each set using either visual assessment (resulting in a perfect ranking) or using the order of a closely related auxiliary variable (resulting in an imperfect ranking), and collect response from them. Let $r_{\left(i\right)}$; i=1,...,ν shows the number of responses obtained for the $i^{th}$ order statistics, then $r-r_{\left(i\right)}$ shows number of no-responding units in the $i^{th}$ order statistics, where *r* is the total number of units in $i^{th}$ order statistics. The formula below is used to calculate the mean of responding units for $i^{th}$ order statistics:

(20) $${\overset{¯}{y}}_{\left(i\right)}=\frac{1}{rP_{\left(i\right)}}\sum_{j=1}^{r_{\left(i\right)}}Y_{j\left(i\right)},$$

where $Y_{j\left(i\right)}$ shows the $j^{th}$ unit for the $i^{th}$ order statistics. $P_{\left(i\right)}$ is the probability of obtaining a response for the $i^{th}$ order statistics. The variance of ${\overset{¯}{y}}_{\left(i\right)}$ is given by

(21) $$V\left({\overset{¯}{y}}_{\left(i\right)}\right)=\frac{1}{rP_{\left(i\right)}}\left(\sigma^{2}-\Delta_{\left(i\right)}^{2}\right).$$

When ${\overset{¯}{y}}_{\left(i\right)}$ is imputed in place of missing values of $i^{th}$ order statistics, the study variable takes the following form

(22) $$Y_{\cdot j(i)}=\left\{ \begin{array}{cc} Y_{j(i)} & \;\text{if }j\in R\text{ with probability }P_{\left(i\right)}\\ {\overset{¯}{y}}_{\left(i\right)} & \;\text{if }j\in \overset{¯}{R}\text{ with probability }(1-P_{\left(i\right)}) \end{array}\right.$$

This leads to propose the first estimator of finite population mean as

(23) $${\overset{¯}{y}}_{Z_{1}}=\frac{1}{r\nu }\sum_{i=1}^{\nu }\left(\sum_{j\in R}Y_{j\left(i\right)}+\sum_{j\in \overset{¯}{R}}{\overset{¯}{y}}_{\left(i\right)}\right)$$

or

(24) $${\overset{¯}{y}}_{Z_{1}}=\frac{1}{r\nu }\sum_{i=1}^{\nu }\left(\sum_{j=1}^{r_{\left(i\right)}}Y_{j\left(i\right)}+\left(r-r_{\left(i\right)}\right){\overset{¯}{y}}_{\left(i\right)}\right).$$

This method involves the imputation of the mean of an order statistic into missing cells of the same order statistic, unlike conventional imputation techniques, where the overall sample mean is imputed for every missing observation. The proposed estimator ${\overset{¯}{y}}_{Z_{1}}$ is unbiased, whereas its variance is given by

(25) $$V\left({\overset{¯}{y}}_{Z_{1}}\right)=\frac{\sigma^{2}}{r\nu^{2}}\sum_{i=1}^{\nu }\left(P_{\left(i\right)}-\frac{{\left(1-P_{\left(i\right)}\right)}^{2}}{P_{\left(i\right)}}\right)-\frac{1}{r\nu^{2}}\sum_{i=1}^{\nu }\Delta_{\left(i\right)}^{2}\left(P_{\left(i\right)}-\frac{{\left(1-P_{\left(i\right)}\right)}^{2}}{P_{\left(i\right)}}\right)\;+\frac{2}{r^{2}\nu^{2}}\sum_{k<i}\sum_{i}\sigma_{\left(k,i\right)}$$

Where $P_{\left(i\right)}=\frac{r_{\left(i\right)}}{r}$, $rP_{\left(i\right)}≅m_{1}$, and rν=m. The term $\sigma_{\left(k,i\right)}$ shows covariance between the $k^{th}$ and $i^{th}$ order statistics such that k<i.

If ranking is based on a closely related auxiliary variable *X*, then the corresponding estimator of the population mean and its variance is given by

(26) $${\overset{¯}{y}}_{Z_{1}}=\frac{1}{r\nu }\sum_{i=1}^{\nu }\left(\sum_{j=1}^{r_{\left[i\right]}}Y_{j[i]}+\left(r-r_{\left[i\right]}\right){\overset{¯}{y}}_{\left[i\right]}\right)$$

and

(27) $$V\left({\overset{¯}{y}}_{Z_{1}}\right)=\frac{\sigma^{2}}{r\nu^{2}}\sum_{i=1}^{\nu }\left(P_{\left[i\right]}-\frac{{\left(1-P_{\left[i\right]}\right)}^{2}}{P_{\left[i\right]}}\right)-\frac{1}{r\nu^{2}}\sum_{i=1}^{\nu }\Delta_{\left[i\right]}^{2}\left(P_{\left[i\right]}-\frac{{\left(1-P_{\left[i\right]}\right)}^{2}}{P_{\left[i\right]}}\right)+\frac{2}{r^{2}\nu^{2}}\sum_{k<i}\sum_{i}\sigma_{\left[k,i\right]}$$

**Figure 1:**
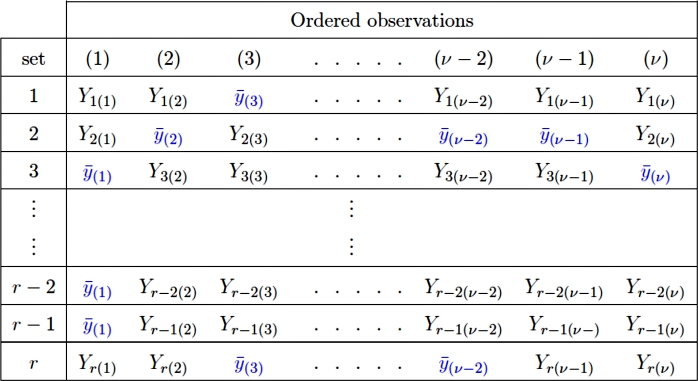
Sampling scheme and proposed imputation method I.

Fig. 1 shows an example of the proposed mean imputation method, where $Y_{j(i)}$ shows the response obtained from $j^{th}$ unit of the $i^{th}$ order statistics, whereas ${\overset{¯}{y}}_{\left(i\right)}$ shows the imputed mean of $i^{th}$ order statistics in place of the missing observations.

The entire process of the first proposed imputation is summarized in the Algorithm 1 given below.

**Algorithm 1:**
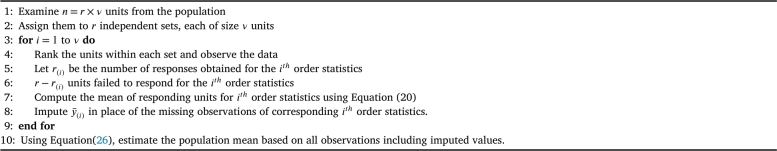
Procedure for estimating population mean based on proposed imputation method I.

### 4.2 Proposed method II

This method differs from the proposed method I in a sense that it employs the RSS technique to choose the units in each set of initial sample. Units within each set are independent, and there is zero covariance between them. This method examines $r\nu^{2}$ units to select a sample of size *rν*. The procedure for this method is described as follows.

Initially, follow the steps explained in Section 2.1 and select *ν* units, place them in the first set. Repeat this procedure *r* times to obtain *r* sets consisting of *ν* ordered units each and collect the response. Let $r_{\left(i\right)}$; i=1,...,ν shows the number of responses obtained for $i^{th}$ order statistics, then $r-r_{\left(i\right)}$ shows the number of non-responding units for the $i^{th}$ order statistics, where *r* shows the total number of units in the $i^{th}$ order statistics. The mean of responding units for $i^{th}$ order statistics is computed as

(28) $${\overset{¯}{y}}_{rss\left(i\right)}=\frac{1}{rP_{\left(i\right)}}\sum_{j=1}^{r_{\left(i\right)}}Y_{i\left(i\right)j},$$

where $Y_{i\left(i\right)j}$ shows the $i^{th}$ order statistics in the $i^{th}$ set of RSS procedure of $j^{th}$ cycle. $P_{\left(i\right)}$ is probability of obtaining response from the $i^{th}$ order statistics. Variance of ${\overset{¯}{y}}_{rss\left(i\right)}$ is given by

(29) $$V\left({\overset{¯}{y}}_{rss\left(i\right)}\right)=\frac{1}{rP_{\left(i\right)}}\left(\sigma^{2}-\Delta_{\left(i\right)}^{2}\right).$$

The study variable in the *ith* order statistics takes the following form

(30) $$Y_{\cdot i(i)j}=\left\{ \begin{array}{cc} Y_{i(i)j} & \;\text{if }j\in R\text{ with probability }P_{\left(i\right)}\\ {\overset{¯}{y}}_{rss\left(i\right)} & \;\text{if }j\in \overset{¯}{R}\text{ with probability }(1-P_{\left(i\right)}) \end{array}\right.$$

This leads to suggest the following estimator of the population mean

(31) $${\overset{¯}{y}}_{Z_{2}}=\frac{1}{r\nu }\sum_{i=1}^{\nu }\left(\sum_{j\in R}Y_{i\left(i\right)j}+\sum_{j\in \overset{¯}{R}}{\overset{¯}{y}}_{rss\left(i\right)}\right)$$

or

(32) $${\overset{¯}{y}}_{Z_{2}}=\frac{1}{r\nu }\sum_{i=1}^{\nu }\left(\sum_{j=1}^{r_{\left(i\right)}}Y_{i\left(i\right)j}+\left(r-r_{\left(i\right)}\right){\overset{¯}{y}}_{rss\left(i\right)}\right).$$

The estimator ${\overset{¯}{y}}_{Z_{2}}$ is unbiased with variance given by

(33) $$V\left({\overset{¯}{y}}_{Z_{2}}\right)=\frac{\sigma^{2}}{r\nu^{2}}\sum_{i=1}^{\nu }\left(P_{\left(i\right)}-\frac{{\left(1-P_{\left(i\right)}\right)}^{2}}{P_{\left(i\right)}}\right)-\frac{1}{r\nu^{2}}\sum_{i=1}^{\nu }\Delta_{\left(i\right)}^{2}\left(P_{\left(i\right)}-\frac{{\left(1-P_{\left(i\right)}\right)}^{2}}{P_{\left(i\right)}}\right)$$

In case of imperfect ranking, the mean estimator and its variance are given by

(34) $${\overset{¯}{y}}_{Z_{2}}=\frac{1}{r\nu }\sum_{i=1}^{\nu }\left(\sum_{j=1}^{r_{\left[i\right]}}Y_{i[i]j}+\left(r-r_{\left[i\right]}\right){\overset{¯}{y}}_{rss[i]}\right)$$

and

(35) $$V\left({\overset{¯}{y}}_{Z_{2}}\right)=\frac{\sigma^{2}}{r\nu^{2}}\sum_{i=1}^{\nu }\left(P_{\left[i\right]}-\frac{{\left(1-P_{\left[i\right]}\right)}^{2}}{P_{\left[i\right]}}\right)-\frac{1}{r\nu^{2}}\sum_{i=1}^{\nu }\Delta_{\left[i\right]}^{2}\left(P_{\left[i\right]}-\frac{{\left(1-P_{\left[i\right]}\right)}^{2}}{P_{\left[i\right]}}\right).$$

**Figure 2:**
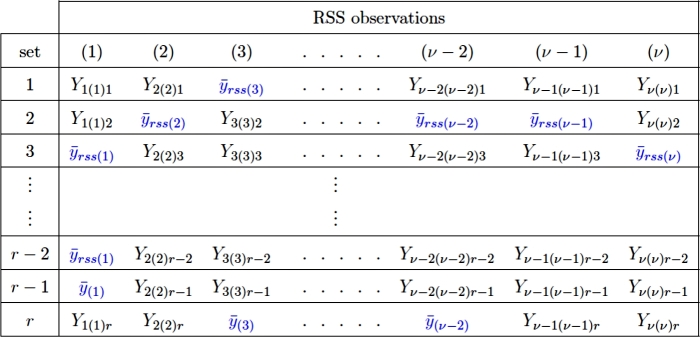
Sampling scheme and proposed imputation method II.

An example of the suggested sampling method and the associated mean imputation is provided in the Fig. 2, where $Y_{i(i)j}$ shows that response is obtained from the $i^{th}$ order statistics in the $j^{th}$ cycle of RSS, whereas the term ${\overset{¯}{y}}_{rss(i)}$ shows imputed mean of the $i^{th}$ order statistics in place of the missing observations.

The Algorithm 2 given below outlines the entire process of the proposed imputation method II.

**Algorithm 2:**
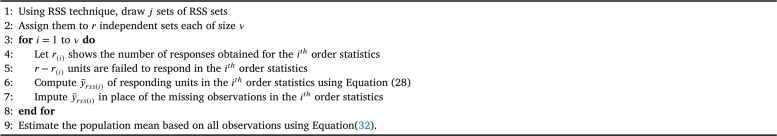
Procedure for estimating population mean based on proposed imputation method II.

### 4.3 Efficiency comparison

If the following conditions are satisfied by the population under study, then the mean estimator under proposed imputation method I will be more precise than

1. the mean estimator under naïve imputation method, if
  $\frac{\sigma^{2}\left(\sum_{i=1}^{\nu }A_{\left(i\right)}-\frac{m\nu }{m_{1}}\right)+2\sum_{k<j}\sum_{j}\sigma_{\left(k,j\right)}}{\sum_{i=1}^{\nu }\Delta_{\left(i\right)}^{2}}<1$.
2. the mean estimator under RSS imputation method, if $\frac{\sigma^{2}\left(\sum_{i=1}^{\nu }A_{\left(i\right)}-\frac{1}{mP}\right)-\sum_{i=1}^{\nu }\Delta_{\left(i\right)}^{2}A_{\left(i\right)}}{\frac{1}{P^{2}}\sum_{i=1}^{\nu^{\prime }}\Delta_{\left(i\right)}^{2}-\frac{2\nu }{m}\sum_{k<j}\sum_{j}\sigma_{\left(k,j\right)}}<1$.
3. the mean estimator under MRSS imputation method with odd *ν*,
  $$\left\{ \begin{array}{cc} \frac{\sigma^{2}\sum_{i=1}^{\nu }A_{\left(i\right)}+\frac{2}{r}\sum_{k<j}\sum_{j}\sigma_{\left(k,j\right)}}{B\sigma_{\left(\frac{\nu +1}{2}\right)}^{2}+\sum_{i=1}^{\nu }\Delta_{\left(i\right)}^{2}A_{\left(i\right)}}<1 & \text{if}\;\text{ν}\;\text{is odd}\\ \;\frac{\left(\sigma^{2}\sum_{i=1}^{\nu }A_{\left(i\right)}+\frac{2}{r}\sum_{k<j}\sum_{j}\sigma_{\left(k,j\right)y}\right)-\sum_{i=1}^{\nu }\Delta_{\left(i\right)}^{2}A_{\left(i\right)}}{\frac{1}{4r}\left(B_{1}\sigma_{\left(\frac{\nu }{2}\right)}^{2}+B_{2}\sigma_{\left(\frac{\nu +2}{2}\right)}^{2}\right)}<1 & \text{if}\;\text{ν}\;\text{is even} \end{array}\right.$$

Similarly, if the population under study meets the conditions given below, the mean estimator under proposed imputation method I will be more precise than

1. the mean estimator under naïve imputation method, if $\frac{\sigma^{2}\left(\sum_{i=1}^{\nu }A_{\left(i\right)}-\frac{\nu }{P}\right)}{\sum_{i=1}^{\nu }A_{\left(i\right)}\Delta_{\left(i\right)}^{2}}<1$,
2. the mean estimator under RSS imputation method, if $\frac{\sigma^{2}\left(\sum_{i=1}^{\nu }A_{\left(i\right)}-\frac{\nu }{P}\right)}{\sum_{i=1}^{\nu }A_{\left(i\right)}\Delta_{\left(i\right)}^{2}+\frac{1}{P^{2}}\sum_{i=1}^{\nu^{\prime }}\Delta_{\left(i\right)}^{2}}<1$,
3. the mean estimator under MRSS imputation method
  $$\left\{ \begin{array}{cc} \frac{\sigma^{2}\sum_{i=1}^{\nu }A_{\left(i\right)}-\sum_{i=1}^{\nu }\Delta_{\left(i\right)}^{2}A_{\left(i\right)}}{\frac{1}{4}B\sigma_{\left(\frac{\nu +1}{2}\right)}^{2}}<1 & \text{if}\;\text{ν}\;\text{is odd}\\ \;\frac{\sigma^{2}\sum_{i=1}^{\nu }A_{\left(i\right)}-\sum_{i=1}^{\nu }\Delta_{\left(i\right)}^{2}A_{\left(i\right)}}{\frac{1}{4r}\left(B_{1}\sigma_{\left(\frac{\nu }{2}\right)}^{2}+B_{2}\sigma_{\left(\frac{\nu +2}{2}\right)}^{2}\right)}<1 & \text{if}\;\text{ν}\;\text{is even}. \end{array}\right.$$

Where

$A_{\left(i\right)}=P_{\left(i\right)}+\frac{{\left(1-P_{\left(i\right)}\right)}^{2}}{P_{\left(i\right)}}$, $B=nP+\frac{\left(1-P\right)}{P}$, $B_{1}=nP_{1}+\frac{\left(1-P_{1}\right)}{P_{1}}$, and $B_{2}=nP_{2}+\frac{\left(1-P_{2}\right)}{P_{2}}$.

## 5 Simulation study

The following procedure is followed to conduct simulation study:

First, generate a bivariate population (Y,X), where values for the study variable *X* are generated using R software via command $X∼rnorm\left(\mu_{x},\sigma_{x}^{2}\right)$. Values for the study variable *Y* are produced using the relationship $Y=\rho_{yx}X+\varepsilon \sqrt{1-\rho_{yx}^{2}}$, where $\rho_{yx}$ is correlation coefficient and *ε* is error term generated as ε∼rnorm(0,1). Now, assuming fixed response rate (RR), draw a sample of size *m* using procedures of existing and proposed methods. The mean of responding $m_{1}$ units is computed and imputed for missing observations as described for each method. This leads to estimate the overall sample mean based on imputed data. This procedure is repeated 25,000 times to compute the RE of proposed estimators. To observe the effect of sample size on RE, we use different values of set size ν(=4,5,6) and number of sets r(=5,10,15). It should be noted that larger set sizes are not recommended in the theory of RSS. In case of imperfect ranking, we use different values of correlation coefficient, i.e., $\rho_{yx}(=0.50,0.90$) to observe the effect of correlation on ranking. We also observe RE for different response rates, i.e., 60% and 70%. RE of the proposed methods is computed using formula

(36) $$RE\left(t_{p}^{⁎}\right)=\frac{MSE\left(t_{E}^{⁎}\right)}{MSE\left(t_{p}^{⁎}\right)},$$

where $MSE(t_{E}^{⁎})$ shows MSE of any exist estimator, whereas $MSE(t_{P}^{⁎})$ shows MSE of the proposed estimators, where MSE for any estimator $t_{•}^{⁎}$ is computed as

(37) $$MSE(t_{•}^{⁎})=\frac{1}{25,000}\sum_{i=1}^{25000}{\left(t_{•}^{⁎}-\mu_{y}\right)}^{2}$$

The simulation results are given below in Table 1, Table 2, Table 3, Table 4.

**Table 1:** RE of proposed mean estimators in case of normal distribution under perfect ranking.

***RR***	***r***	Existing estimator	Proposed estimator I			Proposed estimator II		
			***ν* = 4**	***ν* = 5**	***ν* = 6**	***ν* = 4**	***ν* = 5**	***ν* = 6**
80%	5	${\overset{¯}{y}}_{Isrs}$	3.560	3.707	3.792	3.675	3.826	3.914
		${\overset{¯}{y}}_{Irss}$	2.039	2.123	2.172	2.104	2.191	2.241
		${\overset{¯}{y}}_{Mrss}$	1.551	1.615	1.652	1.601	1.667	1.705
	10	${\overset{¯}{y}}_{Isrs}$	4.831	5.030	5.145	4.986	5.191	5.311
		${\overset{¯}{y}}_{Irss}$	2.329	2.425	2.481	2.404	2.503	2.561
		${\overset{¯}{y}}_{Mrss}$	2.064	2.149	2.199	2.130	2.218	2.269
	15	${\overset{¯}{y}}_{Isrs}$	7.633	7.947	8.130	7.878	8.203	8.391
		${\overset{¯}{y}}_{Irss}$	3.612	3.761	3.847	3.728	3.882	3.971
		${\overset{¯}{y}}_{Mrss}$	3.059	3.185	3.259	3.157	3.288	3.363

70%	5	${\overset{¯}{y}}_{Isrs}$	2.749	2.863	2.929	2.838	2.955	3.023
		${\overset{¯}{y}}_{Irss}$	1.906	1.985	2.030	1.967	2.048	2.096
		${\overset{¯}{y}}_{Mrss}$	1.241	1.292	1.322	1.281	1.334	1.364
	10	${\overset{¯}{y}}_{Isrs}$	3.165	3.296	3.372	3.267	3.402	3.480
		${\overset{¯}{y}}_{Irss}$	1.810	1.885	1.928	1.868	1.945	1.990
		${\overset{¯}{y}}_{Mrss}$	1.470	1.530	1.566	1.517	1.580	1.616
	15	${\overset{¯}{y}}_{Isrs}$	4.692	4.885	4.998	4.843	5.042	5.158
		${\overset{¯}{y}}_{Irss}$	2.387	2.485	2.543	2.464	2.565	2.624
		${\overset{¯}{y}}_{Mrss}$	2.020	2.103	2.151	2.084	2.170	2.220

**Table 2:** RE of proposed mean estimators in case of normal distribution under imperfect ranking.

			Proposed estimator I						Proposed estimator II					
***RR***	***r***	Existing estimator	ρ=0.50			ρ=0.90			ρ=0.50			ρ=0.90		
			***ν* = 4**	***ν* = 5**	***ν* = 6**	***ν* = 4**	***ν* = 5**	***ν* = 6**	***ν* = 4**	***ν* = 5**	***ν* = 6**	***ν* = 4**	***ν* = 5**	***ν* = 6**
80%	5	${\overset{¯}{y}}_{Isrs}$	3.239	3.373	3.450	3.344	3.481	3.561	3.343	3.481	3.561	3.451	3.593	3.676
		${\overset{¯}{y}}_{Irss}$	1.855	1.931	1.976	1.915	1.994	2.039	1.915	1.993	2.039	1.976	2.058	2.105
		${\overset{¯}{y}}_{Mrss}$	1.411	1.469	1.503	1.456	1.516	1.551	1.456	1.516	1.551	1.503	1.565	1.601
	10	${\overset{¯}{y}}_{Isrs}$	4.395	4.576	4.682	4.537	4.724	4.832	4.537	4.723	4.832	4.682	4.875	4.987
		${\overset{¯}{y}}_{Irss}$	2.119	2.207	2.257	2.187	2.278	2.330	2.187	2.278	2.330	2.258	2.351	2.405
		${\overset{¯}{y}}_{Mrss}$	1.878	1.955	2.000	1.938	2.018	2.065	1.938	2.018	2.065	2.001	2.083	2.131
	15	${\overset{¯}{y}}_{Isrs}$	6.945	7.231	7.397	7.168	7.463	7.635	7.168	7.463	7.635	7.398	7.703	7.880
		${\overset{¯}{y}}_{Irss}$	3.286	3.422	3.501	3.392	3.532	3.613	3.392	3.532	3.613	3.501	3.645	3.729
		${\overset{¯}{y}}_{Mrss}$	2.783	2.898	2.965	2.873	2.991	3.060	2.873	2.991	3.060	2.965	3.087	3.158

70%	5	${\overset{¯}{y}}_{Isrs}$	2.502	2.605	2.665	2.582	2.688	2.750	2.582	2.688	2.750	2.665	2.775	2.839
		${\overset{¯}{y}}_{Irss}$	1.734	1.806	1.847	1.790	1.864	1.907	1.790	1.864	1.907	1.848	1.924	1.968
		${\overset{¯}{y}}_{Mrss}$	1.129	1.176	1.203	1.166	1.214	1.241	1.166	1.214	1.241	1.203	1.253	1.281
	10	${\overset{¯}{y}}_{Isrs}$	2.880	2.999	3.068	2.973	3.095	3.166	2.973	3.095	3.166	3.068	3.195	3.268
		${\overset{¯}{y}}_{Irss}$	1.647	1.715	1.754	1.700	1.770	1.811	1.700	1.770	1.811	1.754	1.827	1.869
		${\overset{¯}{y}}_{Mrss}$	1.337	1.393	1.425	1.380	1.437	1.470	1.380	1.437	1.470	1.425	1.483	1.518
	15	${\overset{¯}{y}}_{Isrs}$	4.269	4.445	4.547	4.406	4.588	4.693	4.406	4.588	4.693	4.548	4.735	4.844
		${\overset{¯}{y}}_{Irss}$	2.172	2.261	2.313	2.242	2.334	2.388	2.242	2.334	2.388	2.314	2.409	2.464
		${\overset{¯}{y}}_{Mrss}$	1.838	1.913	1.957	1.897	1.975	2.020	1.897	1.975	2.020	1.957	2.038	2.085

**Table 3:** RE of proposed mean estimators in case of exponential distribution under perfect ranking.

***RR***	***r***	Existing estimator	Proposed estimator I			Proposed estimator II		
			***ν* = 4**	***ν* = 5**	***ν* = 6**	***ν* = 4**	***ν* = 5**	***ν* = 6**
80%	5	${\overset{¯}{y}}_{Isrs}$	3.034	3.16	3.232	3.132	3.261	3.336
		${\overset{¯}{y}}_{Irss}$	1.738	1.809	1.851	1.793	1.867	1.91
		${\overset{¯}{y}}_{Mrss}$	1.322	1.377	1.408	1.365	1.421	1.453
	10	${\overset{¯}{y}}_{Isrs}$	3.923	4.085	4.178	4.049	4.216	4.313
		${\overset{¯}{y}}_{Irss}$	1.891	1.969	2.015	1.952	2.033	2.08
		${\overset{¯}{y}}_{Mrss}$	1.676	1.745	1.786	1.73	1.801	1.843
	15	${\overset{¯}{y}}_{Isrs}$	5.388	5.643	5.792	5.587	5.851	6.004
		${\overset{¯}{y}}_{Irss}$	2.934	3.055	3.125	3.028	3.153	3.226
		${\overset{¯}{y}}_{Mrss}$	2.485	2.587	2.647	2.564	2.671	2.732

70%	5	${\overset{¯}{y}}_{Isrs}$	2.586	2.693	2.755	2.67	2.779	2.843
		${\overset{¯}{y}}_{Irss}$	1.481	1.542	1.578	1.528	1.592	1.628
		${\overset{¯}{y}}_{Mrss}$	1.127	1.173	1.2	1.163	1.211	1.239
	10	${\overset{¯}{y}}_{Isrs}$	3.344	3.482	3.561	3.451	3.593	3.676
		${\overset{¯}{y}}_{Irss}$	1.612	1.679	1.717	1.664	1.733	1.773
		${\overset{¯}{y}}_{Mrss}$	1.429	1.487	1.522	1.474	1.535	1.571
	15	${\overset{¯}{y}}_{Isrs}$	4.592	4.81	4.936	4.762	4.987	5.117
		${\overset{¯}{y}}_{Irss}$	2.501	2.604	2.663	2.581	2.688	2.749
		${\overset{¯}{y}}_{Mrss}$	2.118	2.205	2.256	2.186	2.276	2.328

**Table 4:** RE of proposed mean estimators in case of exponential distribution under imperfect ranking.

			Proposed estimator I						Proposed estimator II					
***RR***	***r***	Existing estimator	ρ=0.50			ρ=0.90			ρ=0.50			ρ=0.90		
			***ν* = 4**	***ν* = 5**	***ν* = 6**	***ν* = 4**	***ν* = 5**	***ν* = 6**	***ν* = 4**	***ν* = 5**	***ν* = 6**	***ν* = 4**	***ν* = 5**	***ν* = 6**
80%	5	${\overset{¯}{y}}_{Isrs}$	2.791	2.907	2.973	2.894	3.015	3.083	2.919	3.040	3.109	2.997	3.120	3.192
		${\overset{¯}{y}}_{Irss}$	1.599	1.664	1.703	1.658	1.726	1.766	1.671	1.740	1.780	1.716	1.786	1.827
		${\overset{¯}{y}}_{Mrss}$	1.216	1.267	1.295	1.261	1.314	1.343	1.272	1.325	1.354	1.306	1.360	1.390
	10	${\overset{¯}{y}}_{Isrs}$	3.609	3.758	3.844	3.743	3.897	3.986	3.774	3.930	4.020	3.874	4.034	4.127
		${\overset{¯}{y}}_{Irss}$	1.740	1.811	1.854	1.804	1.878	1.922	1.819	1.895	1.939	1.868	1.945	1.990
		${\overset{¯}{y}}_{Mrss}$	1.542	1.605	1.643	1.599	1.665	1.704	1.613	1.679	1.718	1.655	1.723	1.763
	15	${\overset{¯}{y}}_{Isrs}$	4.957	5.192	5.329	5.140	5.383	5.526	5.208	5.454	5.596	5.346	5.598	5.745
		${\overset{¯}{y}}_{Irss}$	2.699	2.811	2.875	2.799	2.914	2.981	2.822	2.939	3.007	2.897	3.017	3.087
		${\overset{¯}{y}}_{Mrss}$	2.286	2.380	2.435	2.371	2.468	2.525	2.390	2.490	2.546	2.453	2.556	2.614

70%	5	${\overset{¯}{y}}_{Isrs}$	2.379	2.478	2.535	2.467	2.569	2.628	2.489	2.590	2.650	2.555	2.659	2.720
		${\overset{¯}{y}}_{Irss}$	1.363	1.419	1.452	1.413	1.471	1.505	1.424	1.484	1.517	1.462	1.523	1.558
		${\overset{¯}{y}}_{Mrss}$	1.037	1.079	1.104	1.075	1.119	1.145	1.084	1.129	1.155	1.113	1.159	1.185
	10	${\overset{¯}{y}}_{Isrs}$	3.076	3.203	3.276	3.190	3.322	3.397	3.217	3.349	3.426	3.302	3.438	3.517
		${\overset{¯}{y}}_{Irss}$	1.483	1.545	1.580	1.538	1.602	1.638	1.551	1.615	1.653	1.592	1.658	1.696
		${\overset{¯}{y}}_{Mrss}$	1.315	1.368	1.400	1.363	1.419	1.452	1.374	1.431	1.464	1.410	1.469	1.503
	15	${\overset{¯}{y}}_{Isrs}$	4.225	4.425	4.541	4.381	4.589	4.709	4.439	4.648	4.770	4.556	4.771	4.896
		${\overset{¯}{y}}_{Irss}$	2.301	2.396	2.450	2.386	2.484	2.541	2.406	2.505	2.562	2.469	2.572	2.630
		${\overset{¯}{y}}_{Mrss}$	1.949	2.029	2.076	2.021	2.104	2.152	2.038	2.121	2.170	2.092	2.178	2.227

Simulation results show that the proposed mean estimators under the suggested imputation method are more efficient than the mean estimators under other existing methods. Table 1, Table 2, Table 3, Table 4 show that the RE of the proposed estimators increases with an increase in set size *ν* and overall sample size. The RE also increases as the response rate (RR) is increased. The RE is also observed to be higher in cases of perfect ranking as compared to imperfect ranking. In cases of imperfect ranking, the RE is increased when the association between the study variable and the auxiliary variable is increased. The RE of the mean estimator based on proposed method II is slightly higher than that of proposed method I. This is due to the fact that proposed method II examines more units to select a sample as compared to proposed method I.

## 6 Applications to real data

We consider abalone data originally collected by [15]. Our goal is to estimate the population mean for the shucked weight of abalone, which is the total weight of meat after the shell is removed. We assume that non-response occurs in the data due to the evasive nature of abalone, which can make sampling challenging. The study variable *Y* and the auxiliary variable *X* are taken as

*Y* = Shucked weight of abalone in grams (continuous)

*X* = Height of abalone in millimeters (continuous)

Simulation procedure is the same as described in Section 5. The summary statistics of variables *Y* and *X*, and the RE of the mean estimator under the proposed methods are given in the Table 5, Table 6, respectively. The simulation procedure is same as described in Section 5. RE of the mean estimator under proposed methods is given in below tables.

**Table 5:** Descriptive statistics of the data.

Variable	*N*	*Minimum*	*Q*_1_	*Q*_2_	*Mean*	*Q*_3_	*Maximum*
***Y***	4175	0.0020	0.4415	0.7995	0.8287	1.1530	2.8255
***X***	4175	0.0100	0.1150	0.1400	0.1395	0.1650	1.1300

**Table 6:** RE of proposed mean estimators in case of perfect ranking.

*RR*	*r*	Existing estimator	Proposed estimator I			Proposed estimator II		
			***ν* = 4**	***ν* = 5**	***ν* = 6**	***ν* = 4**	***ν* = 5**	***ν* = 6**
80%	5	${\overset{¯}{y}}_{Isrs}$	3.038	3.164	3.236	3.136	3.265	3.340
		${\overset{¯}{y}}_{Irss}$	1.740	1.812	1.854	1.796	1.870	1.912
		${\overset{¯}{y}}_{Mrss}$	1.324	1.378	1.410	1.366	1.423	1.455
	10	${\overset{¯}{y}}_{Isrs}$	4.123	4.293	4.391	4.255	4.430	4.532
		${\overset{¯}{y}}_{Irss}$	1.988	2.069	2.117	2.052	2.136	2.186
		${\overset{¯}{y}}_{Mrss}$	1.761	1.834	1.877	1.818	1.893	1.936

70%	5	${\overset{¯}{y}}_{Isrs}$	2.346	2.443	2.500	2.422	2.522	2.580
		${\overset{¯}{y}}_{Irss}$	1.627	1.694	1.732	1.679	1.748	1.789
		${\overset{¯}{y}}_{Mrss}$	1.059	1.103	1.128	1.093	1.138	1.164
	10	${\overset{¯}{y}}_{Isrs}$	2.701	2.813	2.878	2.788	2.903	2.970
		${\overset{¯}{y}}_{Irss}$	1.545	1.609	1.645	1.594	1.660	1.698
		${\overset{¯}{y}}_{Mrss}$	1.254	1.306	1.336	1.295	1.348	1.379

## 7 Conclusions

Non-response is a critical issue faced by surveyors when conducting surveys; this results in missing data. Although omitting missing observations is a common practice in statistical analysis, it reduces the sample size, which is critical for obtaining precise statistical estimates. One method of dealing with missing data is imputation of the mean for missing observations. In this paper, we proposed two new estimators of the finite population mean, which are based on two suggested sampling methods to be used in surveys anticipating non-response. Expressions are derived for the variance of proposed estimators, and a theoretical efficiency comparison with other existing estimators is provided. A simulation study based on two hypothetical populations and abalone data is conducted for varying sample sizes and ranking criteria to observe the relative efficiency (RE) of the proposed estimators. The simulation findings showed that the proposed estimators are more efficient than existing estimators. Based on our study, we recommend the use of proposed survey methods and the associated mean estimators when non-response is anticipated from the respondents. The work presented in this paper on mean imputation can serve as a basis for developing more advanced imputation methods like ratio and regression.

## Funding disclosure

This research was financially supported by Prince Nourah bint Abdulrahman University Researchers Supporting Project number PNURSP2024R443, Princess Nourah bint Abdulrahman University, Riyadh, Saudi Arabia.

## Declaration of generative AI and AI-assisted technologies in the writing process

No AI or AI-assisted technologies were used in the writing, research, or preparation of this manuscript. The work presented herein is the result of human effort and expertise without any computational assistance from AI systems.

## CRediT authorship contribution statement

**Syed Abdul Rehman:** Writing – original draft, Conceptualization. **Javid Shabbir:** Methodology. **Laila A. Al-essa:** Software, Funding acquisition, Data curation.

## Declaration of Competing Interest

The authors declare the following financial interests/personal relationships which may be considered as potential competing interests: Laila A. Al-essa reports a relationship with Prince Nourah bint Abdulrahman University Researchers Supporting Project that includes: employment. If there are other authors, they declare that they have no known competing financial interests or personal relationships that could have appeared to influence the work reported in this paper.

## Data Availability

The data for this study was obtained from the UCI Machine Learning Data Repository accessed at https://archive.ics.uci.edu/dataset/1/abalone.
